# Editorial: Plant-bacteria association and symbiosis

**DOI:** 10.3389/fpls.2024.1423947

**Published:** 2024-05-10

**Authors:** Carlos Henrique S. G. Meneses, Diogo Neves Proença, German A. Estrada-Bonilla, Marcia Soares Vidal

**Affiliations:** ^1^ Laboratory Plant Biotechnology, Graduate Program in Agricultural Sciences, Department of Biology, Center for Biological and Health Sciences, State University of Paraíba, Campina Grande, Brazil; ^2^ Department of Life Sciences, Centre for Mechanical Engineering, Materials and Processes (CEMMPRE), Advanced Production and Intelligent Systems (ARISE), University of Coimbra, Coimbra, Portugal; ^3^ Agricultural Microbiology Laboratory, Tibaitatá Research Center, Corporación Colombiana de Investigación Agropecuaria (AGROSAVIA), Mosquera, Colombia; ^4^ Laboratory of Genetics and Biochemistry, Empresa Brasileira de Pesquisa Agropecuária – EMBRAPA, Embrapa Agrobiologia, Rio de Janeiro, Seropédica, Brazil

**Keywords:** plant-bacteria interactions, symbiosis, plant growth-promoting bacteria, endophytes, epiphytes

In the relentless pursuit of sustainable agricultural practices, society has pivoted its gaze towards alternatives to synthetic chemical fertilizers, recognizing the significant environmental impact they impose. Among the myriad of alternatives, the use of plant growth-promoting bacteria (PGPB) has emerged as a promising solution, encouraging potential to revolutionize plant nutrition in a manner that is both effective and environmentally sustainable. The interaction between plants and PGPB is a wonder of nature, encompassing a wide array of interactions that extend far beyond simple nutrient provision. These remarkable microorganisms, through their ability to harness unavailable nutrients and synthesize essential phytohormones, exert a profound influence on plant metabolism, enhancing growth and resilience even in challenging conditions.

At the heart of the challenge lies the enigmatic nature of plant-microbe interactions, fraught with complexities that confound even the most seasoned researchers. The quest to elucidate the dynamic interplay between plants and microorganisms across diverse environmental conditions remains a formidable task, yet one that is essential for unlocking the full potential of PGPB in sustainable agriculture. In their tireless quest for knowledge, researchers have harnessed the power of omics technologies to decipher the intricate network of biochemical, genetic, genomic, and molecular interactions that underpin the symbiotic relationship between plants and bacteria. However, despite the progress made, many mysteries remain unsolved, with intriguing discoveries awaiting exploration.

In our unwavering commitment to advancing crop improvement and fostering sustainable agriculture, we are proud to present a Research Topic dedicated to unraveling the mysteries of plant-bacteria relationships. The current Research Topic includes one review, one brief research report article and 10 original research studies focusing on (i) selection of efficient microbial strains and their characterization regarding their potential to alleviate abiotic stress; (ii) utilizing effective microbial species to enhance soil nutrient availability for plants; (iii) exploring bacterial diversity, including both plant growth-promoting bacteria in cultivated plants and rhizospheric soil, and on (iv) describes the importance of a bacterial biopolymer in the plant intracellular colonization process.

The review presented by Choudhary et al. address the diversity of endophytic microorganisms and their significance in various industries, including agricultural, pharmaceuticals and biotechnology. Specifically, the authors highlight the importance of these endophytes in producing bioactive compounds with pharmaceutical and agricultural applications.

Regarding the first topic presented, da Silva et al. report on the responses of nitrogen fixation in bean nodules during drought, emphasizing the regulation of heat shock proteins and transcription factors. Meanwhile, Kim et al. demonstrate enhanced drought resistance in *Arabidopsis* and *Brassica* plants using the *Flavobacterium* sp. GJW24. Likewise, dos Santos et al. explore the molecular and biochemical responses of sesame to rhizobacteria inoculation under drought conditions. Finally, Teles et al. focus on the characterizing and evaluating halotolerant phosphate-solubilizing bacteria from the rhizosphere of *Salicornia fruticosa*. In the context of climate change, characterized by increasingly severe drought periods, these studies provide valuable insights for understanding and advancing agriculture under challenging conditions, including drought and salinity.

In the second topic of study, Tounsi-Hammami et al. optimize the growth of tomato seedlings by inoculation of indigenous mangrove bacteria and potentially reduce NPK fertilization. Results showed that applying bacterial inoculant with 50% NPK significantly increase plant growth, photosynthetic activity, and nutrient uptake. In another study, Rosman et al. new insights into on the relationship between plants, plant growth-promoting bacteria, and the environment. Furthermore, Nagah et al. demonstrate that endophytic bacteria isolated from medicinal plants enhance phosphorus acquisition, leading to improve vegetative growth and metabolic content in *Brassica napus* L. Lastly, Lu et al. evidence of increased phosphate uptake and alterations in bacterial communities in maize rhizosphere soil attributed to the presence of arbuscular mycorrhizal fungi. These studies contribute to the field plant-microbe-environment interactions, highlighting the significance of symbiotic relationships for crop growth and development.

In the third topic of research, Gätjens-Boniche et al. explore the connection between the microbiome of insect-induced galls in cassava and the genetic transformation of plant cells. Results indicate that microbiome enrichment and genetic transformation of plant cells contribute to gall development. Meanwhile, Shi et al. address the characteristics of the phyllosphere microbial community and its relationship to key aroma precursors during the tobacco maturation process. Both the study delves into various aspects of bacterial diversity, including their role in gall development in cassava and their influence on aroma precursor production during tobacco maturation.

Concluding the Research Topic, Baruah et al. present findings concerning the mobilization of poly-3-hydroxybutyrate and its significance in reducing protein aggregation in *Methylorubrum extorquens* DSM1306 under oxidative stress. The authors illustrate the pivotal role of biopolymer in initial stages of intracellular colonization.

In conclusion, the diverse range of research presented in this Research Topic underscores the pivotal role of plant-microbe interactions, especially the PGPB participation, in advancing sustainable agricultural practices. From elucidating the biochemical and molecular mechanisms underlying these interactions to exploring the potential of microbial inoculants in alleviating abiotic stresses and enhancing nutrient availability, each study contributes with valuable insights that pave the way for a greener and more resilient agricultural future. Additionally, the findings highlight the intricate dynamics between plants, microorganisms, and their environment, with the significance of symbiotic relationships in shaping crop growth and development under challenging conditions such as drought and salinity stresses. As we continue to unlock the secrets of sustainable agriculture, the synergistic partnership between plants and microorganisms emerges as a cornerstone for achieving a more sustainable and prosperous future for generations to come.

## Author contributions

CM: Conceptualization, Writing – original draft, Writing – review & editing. DP: Conceptualization, Writing – original draft, Writing – review & editing. GE-B: Conceptualization, Writing – original draft, Writing – review & editing. MV: Conceptualization, Writing – original draft, Writing – review & editing.

